# Management of Pregnancy in Women with Inflammatory Bowel Disease: Positioning Janus Kinase Inhibitors Within Current Evidence

**DOI:** 10.3390/cimb48040421

**Published:** 2026-04-19

**Authors:** Dario Colacurci, Raffaele Pellegrino, Alessia Lamart, Davide Staiano, Ilaria De Costanzo, Michele Izzo, Giuseppe Imperio, Fabio Landa, Giulia Scamardella, Enrica Di Lella, Alessandro Federico, Laura Sarno, Antonietta Gerarda Gravina

**Affiliations:** 1 Department of Public Health, School of Medicine, University of Naples Federico II, 80131 Naples, Italy; 2 Hepatogastroenterology Division, Department of Precision Medicine, University of Campania Luigi Vanvitelli, 80138 Naples, Italy; 3 Department of Neuroscience, Reproductive Sciences and Dentistry, University of Naples Federico II, 80131 Naples, Italy; 4 San Giuliano Hospital, 80014 Giugliano in Campania, Italy

**Keywords:** inflammatory bowel disease, pregnancy, Janus kinase inhibitors, tofacitinib, upadacitinib, pregnancy outcomes, disease activity, placental transfer, breastfeeding, clinical management

## Abstract

Inflammatory bowel diseases (IBD) frequently affect women of reproductive age. Disease activity may arise during pregnancy, at times in severe forms, thereby generating complex clinical scenarios. Adequate control of disease activity throughout pregnancy and the achievement of a safe delivery with a healthy newborn, therefore, represent vital objectives in therapeutic management. In recent years, the therapeutic armamentarium for moderate to severe IBD has expanded exponentially, with the introduction of biological agents and small molecules. However, although these therapies have largely superseded conventional treatment in complex settings, they do not share the same safety profile in pregnancy. Concerns persist regarding potential transplacental transfer and possible teratogenic effects, which justify mandatory caution in their use during pregnancy. Nonetheless, clinicians may readily encounter scenarios of active IBD during pregnancy in patients who have previously experienced failure of the biological agents most extensively studied in this context, thus necessitating an evaluation of the safety of more novel therapeutic options. This review examines the available evidence on Janus kinase inhibitors. Current data, which are highly heterogeneous and of low quality, preclude any recommendation for the use of these small molecules during pregnancy. Prospective registries and large-scale observational studies are mandatory, pending the feasibility of dedicated trials, to better characterise these inhibitors, which could prove valuable, should the evidence ultimately support their use, in women with biologic multi-failure active IBD during pregnancy.

## 1. General Considerations on Pregnancy in Patients with Inflammatory Bowel Disease

Crohn’s disease (CD) and ulcerative colitis (UC) are part of a spectrum of diseases defined as Inflammatory Bowel Disease (IBD) [1]. IBD predominantly affects women during reproductive age [2]. The IBD spectrum is associated with fertility and pregnancy complications that are clinically significant [3]. Management of IBD during pregnancy remains a complex problem, largely based on limited available data, despite recent therapeutic advances [4]. Considering the uncertainty surrounding the management of pregnant women with IBD, drug suspension is common in clinical practice. However, this can worsen the disease, which is the major determinant of pregnancy outcomes [5]. There is also a lack of awareness of patients’ knowledge of IBD and pregnancy. The “Crohn’s and Colitis Pregnancy Knowledge Score” is a powerful tool for assessing awareness [6], and studies show that almost half of patients have poor knowledge of fertility, pregnancy, and drug safety. This also contributes to increasing rates of voluntary childlessness among IBD patients [7]. In recent decades, there has been a significant increase in large cohorts and international databases [8,9,10]. This literature has helped develop consensus-based recommendations. Indeed, worldwide initiatives have defined structured methodologies to provide standardised guidelines [11,12]. The authors highlight the importance of individualised, multidisciplinary, and patient-centred care during the preconception, antenatal, and postpartum periods. Sexual function and fecundity can be affected by systemic inflammation and disease manifestations. Patients with active IBD, especially those presenting with perianal disease, experience sexual dysfunction [13,14]. These issues can affect quality of life and fecundity. Moreover, the fear of heritability is the main barrier to parenthood. However, the heritability risk is still low, and appropriate counselling appears to improve reproductive decisions and reduce unnecessary childlessness [15]. On the other hand, disease activity can negatively influence ovarian reserve, thereby affecting fecundity [16]. Flares can reduce conception rates and anti-Müllerian hormone levels. Moreover, surgeries, including ileal pouch-anal anastomosis (IPAA) and proctectomy, can cause infertility, mainly due to the development of pelvic adhesions [4].

Assisted reproductive technology is effective for women with well-controlled disease. However, the success rate can be lower for women with CD and previous bowel surgery [17]. Disease activity, nutritional status, and micronutrient deficiencies must be adequately assessed, especially during the preconception period. Iron, vitamin D, and folate levels must be sufficient. Anaemia is extremely frequent in IBD patients, and it is frequently not diagnosed and not treated, regardless of its benefits on maternal and neonatal outcomes. Moreover, clinicians consider anaemia as a marker of insufficient disease control. Active inflammation, impaired absorption, and chronic intestinal blood loss represent the most common causes of iron deficiency and anaemia, particularly in chronic gastrointestinal diseases. In obstetric care, anaemia is a major risk factor for postpartum haemorrhage; indeed, patients suffering from it have an increased rate of blood transfusions, delayed wound healing, and prolonged postpartum recovery.

Considering this, routine screening for haemoglobin and iron parameters should be incorporated into preconception and antenatal care for IBD patients. Nevertheless, anaemia in IBD patients may increase thromboembolic risk through secondary thrombocytosis and platelet activation, increasing the prothrombotic state of pregnancy and the postpartum period. Recent studies [18] support early correction of iron deficiency. Intravenous formulations are preferable, particularly in patients with active disease, moderate-to-severe anaemia, or intolerance to oral supplementation. Adequate iron levels before delivery may help prevent adverse outcomes due to peripartum blood loss, thereby reducing emergency intervention rates. Lastly, recurrence is common: half of treated patients experience it, so structured follow-up and long-term monitoring are needed. The drug and therapy regimen must be adequately reviewed. Indeed, to avoid teratogenic agents [19].

Optimal prenatal care has been associated with improved pregnancy and neonatal outcomes, as well as reduced disease activity during pregnancy. In IBD patients, the most important predictor of pregnancy outcomes is disease activity at conception [3]. Remission IBD patients have the highest probability of maintaining disease stability. Inflammation stability is significantly related to miscarriage, preterm birth, low birth weight, small-for-gestational-age infants, and intrauterine growth restriction [20]. In addition, maintaining remission is still the first-line therapeutic objective in pregnant patients. Several studies have clearly established that the fertility of patients in remission is comparable to that of the general population. Instead, the fertility of IBD patients is reduced in the presence of inflammation, distress, malnutrition, and after surgery, particularly in patients who have undergone IPAA procedures. An essential step in the management of IBD patients is counselling and preconception care. Even in the absence of a desire to conceive, counselling should be initiated as soon as possible, as unplanned pregnancies are very common. The main aim is to achieve continuous clinical and biochemical remission for at least three to six months prior to conception. Moreover, patients themselves are at risk of complications such as anaemia [21], venous thromboembolism, malnutrition, gestational diabetes, and flare-ups that result in hospitalisation [22].

Systemic inflammation, which is linked to the prothrombotic state of pregnancy, also poses a risk for thromboembolism, especially postpartum. Lastly, pregnancy in an IBD patient should be considered high risk. Management should be carried out by a multidisciplinary team including gastroenterologists and obstetricians, as well as other members such as nutritionists, surgeons, and psychologists when necessary. Some diagnostic tools are available to help clinicians evaluate the physiological changes occurring during pregnancy. In particular, faecal calprotectin can be used to assess disease activity, and bowel ultrasound and magnetic resonance imaging without gadolinium are possible tools for the management of pregnant women with IBD.

Concerning the mode of delivery, vaginal delivery is considered safe for the vast majority of women suffering from IBD and should be the first option when there are no contraindications. Several authors suggested that vaginal delivery worsens perianal CD, but there is no strong evidence to confirm this. One study [23] reported a 17.9% rate of new-onset perianal disease after vaginal delivery, often associated with episiotomy. However, the unclear definition of perianal disease makes it difficult to differentiate true exacerbations from perineal wound complications. Hatch et al. [24] found similar episiotomy rates in CD and non-CD patients without perianal disease, despite recommendations to avoid it in CD. The authors assumed that this likely reflects a general decline in episiotomy use rather than specific caution in CD patients. Due to limited data, it remains unclear whether episiotomy is truly avoided in IBD patients. Severe perineal lacerations are clinically relevant because they are associated with major postpartum morbidity, including faecal incontinence and rectovaginal fistula. The literature is limited in comparing these outcomes in IBD patients. Current evidence [24] suggests similar rates of second- and third-degree lacerations in CD and non-CD women, while fourth-degree tears appear more related to perianal disease than to CD itself. On the other hand, caesarean section should be recommended for patients suffering from active perianal CD or those with a history of rectovaginal fistula or restorative proctocolectomy. Analysing a large, nationally representative US inpatient database [25], IBD remained associated with caesarean delivery (26% higher likelihood), even after adjustment for demographic, medical, and obstetric factors, indicating that this risk cannot be explained solely by conventional obstetric indications. Indeed, authors suggest that unmeasured factors, such as higher risk perception, defensive clinical practice, variability in counselling, and differences in institutional guidelines, may contribute to a progressive medicalisation of childbirth in women with IBD.

On the other hand, the presence of at least one IBD severity criterion (penetrating disease, stricturing disease, perianal disease, history small or large intestine resection, protein-calorie malnutrition, parenteral nutrition, *Clostridioides difficile* colitis, pouchitis or lower gastrointestinal endoscopy) doubled the risk of caesarean section (RR 2.26); this reinforce the idea that cumulative disease features are the main driver of delivery mode, rather than IBD diagnosis alone. These findings promote individualised risk stratification rather than routine surgical delivery. Moreover, surgical injuries were significantly more frequent in women with IBD, probably because of higher rates of technical complexity related to adhesions and previous abdominal procedures.

Finally, IBD-delivery mode should not be a default option, but a carefully weighed decision requiring multidisciplinary assessment and experienced surgical support.

The methods for conducting this narrative review are described in detail in Appendix A.

## 2. The Role of the JAK–STAT Pathway in Pregnancy: Implications for Maternal–Foetal Health

Cytokine-mediated signalling pathways play a key role in regulating pregnancy [26]. Indeed, they seem to modulate the vascular, immune, and endocrine systems to maintain balance from conception to delivery [27]. Among them, the Janus kinase-signal transducer and activator of transcription (JAK-STAT) is one of the regulators in the intracellular signalling cascade that mediates the pathways of cellular proliferation, differentiation, and immune regulation [28]. Authors began studying this pathway as a key regulator of immune system diseases in patients desiring pregnancy.

In particular, JAK-STAT coordinates many cytokines, such as interferons, leukaemia inhibitory factor (LIF), interleukin-6 (IL-6), and interleukin-2 (IL-2) [29], which favour endometrial receptivity and trophoblast invasion during embryo implantation and early placental formation. Studies have shown that the STAT pathway is essential for the early decidualization and the embryo attachment [30]. The inhibition of this pathway could lead to miscarriages with impaired implantation. So, the proper function of JAK-STAT signalling appears to be necessary for the integrity of pregnancy. Indeed, its coordination of several interleukins, which mediate the action of regulatory T cells, natural killer cells, and macrophages, prevents maternal immune activation against the semi-allogenic foetus. So, several obstetric complications are born during this moment of alteration, which is associated with miscarriage, preeclampsia (PE), and foetal growth restriction (FGR). PE and FGR are strictly associated with damage to the vascular endothelium. Endothelial cells generally regulate the vascular tone through vasodilatory and vasoconstrictive factors.

Evidence has shown that the JAK/STAT pathway regulates endothelial cells. Its malfunction could lead to excessive production of metalloproteinases that favour the degradation of endothelial proteins, such as fibronectin and laminin, leading to endothelial cell dysfunction. In PE, an increased rate of pro-inflammatory cells and cytokines leads to the production of placental reactive oxygen species that impair normal exchange between the mother and foetus. This leads to impaired vascular relaxation, trophoblast invasion, and overall endothelial dysfunction [31]. Moreover, the same cytokines and growth factors promote vascular remodelling and angiogenesis to mediate placental development by producing vascular endothelial growth factor and placental growth factor. Indeed, trophoblast cells and placental endothelial cells are rich in STAT proteins that regulate proliferation, migration, and survival. So, it seems clear that JAK inhibitors (JAKi) could alter both maternal immune adaptation and foetal development. As said before, literature is vague, mostly characterised by small case series and case reports, with insufficient data to give proper pathogenesis and aetiology. So, in clinical practice, clinicians personalise JAKi therapies depending on patients’ clinical features and symptoms. Taken together, these mechanisms indicate that any pharmacological interference in the JAK/STAT pathway, like the use of JAKi, has the potential to interfere with key events in pregnancy, including decidualization, trophoblast invasion, immune tolerance, and vascular function. These changes in the JAK/STAT pathway may thus be associated with clinically significant adverse pregnancy outcomes, like miscarriage, preeclampsia, and growth restriction. These mechanistic discussions form a biological basis for understanding the clinical observations presented in the sections that follow. However, these remain potentially speculative considerations, since definitive studies on the phenomena and epiphenomena underlying such potential interactions are not yet fully available.

## 3. Management of IBD During Pregnancy: Insights from the Main IBD-Focused International Guidelines

The management of IBD during pregnancy has been codified by several international IBD-focused guidelines, establishing a model of patient care that begins prior to conception, particularly through thorough preconception counselling, and extends to the appropriate management and monitoring of IBD throughout pregnancy up to delivery.

### 3.1. What Considerations Should Be Addressed Prior to Pregnancy?

Preconception counselling is essential, as the patient should receive adequate information regarding the risk of heritability of IBD in the offspring, the potential adverse events associated with pregnancy in women with immune-mediated diseases, particularly when active, and, finally, the therapeutic and screening measures, for example, for nutritional deficiencies, that should be undertaken in this setting.

The genetic risk of transmission of IBD is tangible. In particular, according to available data, this risk appears to be higher in CD than in UC, with some studies estimating an inter-IBD difference of 2.7% versus 1.6% [11], and others reporting rates of 5% versus 3% [12]. As suggested by ECCO guidelines, this risk seems to increase in the presence of multiple affected family members, younger age at IBD diagnosis, Caucasian ethnicity, and, finally, it is increased by 30% when both parents are affected by IBD.

Disease status in terms of activity has a profound impact on pregnancy outcomes. Indeed, active IBD at the time of conception is associated with concrete foetal risks, including preterm birth [32,33,34,35,36], low birth weight [32,33,34,35,36] and small for gestational age syndromes [34,35,37], as demonstrated by several observational studies.

In addition, in this scenario, patients exhibit a higher frequency of diabetes (odds ratio 2.96, 95% confidence interval 1.47 to 5.98), independent of corticosteroid use, and a greater risk of preterm premature rupture of membranes (odds ratio 12.10, 95% confidence interval 2.15 to 67.98) [38].

As anticipated, there are further considerations regarding the need to implement preventive pharmacological measures, about which women planning pregnancy should be informed in advance. European and American guidelines [12,39] are aligned in recommending a risk-weighted low-dose aspirin for pre-eclampsia prophylaxis from the twelfth week of gestation, to be discontinued close to delivery.

Moreover, they recommend daily folic acid supplementation, with a standard dose of 400 micrograms for the general population and higher doses for at-risk groups, such as patients receiving sulfasalazine, those who have undergone significant intestinal resections, or those with active disease, malabsorption, or a family history of neural tube defects [11,39].

The World Health Organisation recommends oral iron supplementation of 30 to 60 mg per day in all pregnant women; in patients with IBD, the dose increases to 100 mg per day [40]. Where oral iron is not tolerated or proves ineffective, intravenous iron should be considered and reserved for the second and third trimesters after careful evaluation of maternal and foetal risks and benefits; during the first trimester, intravenous administration is generally avoided (unless strongly indicated) because of potential teratogenicity [39,41].

A final, yet no less important, consideration to be discussed with the patient is the need to plan pregnancy in a setting of well-controlled disease. To this end, a global consensus recommends sustained remission for at least three months prior to conception, defined as steroid-free clinical remission with a faecal calprotectin level below 150 micrograms per gram [39]; the 2025 British guidelines also advocate remission for at least three months [11].

If the disease is active, it may be theoretically advisable to postpone pregnancy planning and opt for long-acting reversible contraception not containing oestrogen, such as intrauterine devices or contraceptive implants [39].

### 3.2. What Considerations Should Be Addressed During Pregnancy?

At the onset of pregnancy, appropriate monitoring of IBD should be planned in order to detect potential relapses or to assess the response to treatment when the disease is active at baseline. Such monitoring, which must take into account the potential impact of diagnostic techniques on pregnancy, can be performed non-invasively by measuring faecal calprotectin, whose levels are well known to correlate with disease activity [12].

Conversely, among commonly used inflammatory markers, C-reactive protein should be interpreted with caution, as physiological increases have been reported during pregnancy [42].

More invasive procedures, such as endoscopy, should be reserved for cases in which, after careful risk-benefit assessment, they are deemed mandatory in order to guide clinically relevant medical decisions, preferably after the second trimester [43], using midazolam as the preferred agent when conscious sedation is required [44,45]. The risks of endoscopy include both the procedure itself and bowel preparation. Endoscopy may lead to maternal and foetal hypoxia and to maternal hypotension with consequent reduction in placental perfusion. This risk can be mitigated by positioning the patient tilted to the left or in the left lateral decubitus position, thus avoiding compression of the aorta or the superior vena cava. In addition, attention should be paid to bowel preparation: polyethylene glycol has not been studied in pregnancy and should therefore be avoided; sodium phosphate solution may cause a hydro-electrolyte imbalance and should be used with particular caution [11,12,43,44,45,46,47].

Concerning non-endoscopic cross-sectional radiological imaging techniques, those that do not involve ionising radiation are clearly preferable, such as intestinal ultrasound and magnetic resonance imaging without gadolinium [48,49,50].

Regarding vaccination during pregnancy, international consensus statements agree on the use of inactivated-virus vaccines in pregnant women with IBD, such as influenza and pertussis vaccines [51].

As previously mentioned, clinicians may encounter pregnancies in women with already active IBD, as well as disease flares, including severe ones, in patients whose disease was inactive at preconception baseline [52]. A previous pregnancy complicated by a disease flare and the presence of disease activity at conception are key determinants of the risk of intra-uterine relapse, as shown by Rottenstreich et al. [52].

Hence, the gastroenterologist needs to be familiar with which therapeutic agents from the armamentarium available for the general non-pregnant population can be safely employed in pregnancy, clearly favouring drugs with lower teratogenic potential and lower pregnancy-related risk.

European and American guidelines [12,39] consider mesalazine, sulfasalazine, corticosteroids, thiopurines, and certain biologic agents, such as anti-TNF therapies (including infliximab, adalimumab, and golimumab) [53], as well as vedolizumab [54] and ustekinumab, to be low risk in pregnancy. Surgery remains an option when clinically indicated [12,55,56,57].

Specifically, mesalazine exhibits lower levels in foetal plasma than in maternal plasma [58]. Sulfasalazine, owing to its antifolate activity, should be combined with folic acid supplementation, as previously discussed [12,39].

Thiopurine therapy may be continued, although it is associated with a risk of intrahepatic cholestasis [11,39,53,59,60]. Monitoring of liver enzymes and metabolite levels is required, and dose splitting may be considered [39,60].

According to British guidelines, pregnancy also influences the pharmacokinetics of biologics: adalimumab and ustekinumab show stable plasma levels, whereas infliximab levels increase and vedolizumab levels decrease [11,61]. Foetal levels of biologics are higher than maternal concentrations [61,62,63], yet treatment should not be discontinued, as withdrawal is associated with IBD relapse.

In pregnant women with fistulae or pouchitis, low-risk antibiotics such as metronidazole [64,65,66] or amoxicillin with or without clavulanic acid [67] may be used. Ciprofloxacin should be avoided during the first trimester because of the risk of arthropathy [68].

American guidelines [39] also allow continuation during pregnancy of anti-IL-23 agents, including mirikizumab, risankizumab and guselkumab. In addition, they support the use of calcineurin inhibitors as rescue therapy to avoid colectomy [39,69,70,71,72,73].

Conversely, several therapeutic classes are generally regarded by these consensus statements as potentially teratogenic or unsafe, including methotrexate, JAKi and sphingosine 1 phosphate modulators.

Methotrexate is a folic acid antagonist and is teratogenic; its use in pregnancy is associated with various craniofacial anomalies, including craniosynostosis, musculoskeletal abnormalities, microcephaly and cardiac defects such as tetralogy of Fallot and pulmonary valve atresia [39,74,75,76,77]. Therefore, in anticipation of pregnancy, methotrexate must be discontinued at least three months in advance.

JAKi are small molecules that cross the placenta and, according to American guidelines [39], their use in pregnancy is relatively contraindicated. They may be continued only when they represent the sole therapeutic option capable of controlling the disease [39]. Otherwise, tofacitinib and upadacitinib should be discontinued four weeks before planned conception, and filgotinib one week before [39]. Animal studies have demonstrated possible teratogenicity of tofacitinib at doses 6.3 times higher than those typically used in humans [12,78,79,80]. At doses comparable to those used in humans, filgotinib has been associated with foetal death and severe malformations [39], while upadacitinib has been linked to musculoskeletal and cardiovascular malformations [39,81].

Sphingosine-1-phosphate modulators are likewise considered relatively contraindicated by American guidelines [39]. They should be continued only if no alternative therapeutic option is available. Otherwise, ozanimod should be discontinued at least three months prior to pregnancy and etrasimod one to two weeks before conception. Animal studies have demonstrated an association between foetal death and severe malformations with ozanimod at doses equivalent to those used in humans; a similar association has been observed with etrasimod at doses five to six times higher [39].

The list of drugs considered safe in pregnancy is summarised in Table 1 [65,68,82,83,84,85,86,87,88,89,90,91,92,93,94,95,96,97,98,99,100,101,102,103,104,105,106].

Finally, as already stated, the gastroenterologist may be involved in assessing the safest mode of delivery. Several studies favour vaginal delivery [46], except in cases of perianal disease, ileoanal pouch or ileorectal anastomosis, in which caesarean section is preferred [11]. A systematic review with meta-analysis reported an increased risk of caesarean section in women with IBD, particularly in those with UC [38]. Predictive factors for caesarean section include, in UC, smoking, pancolitis and ileal pouch anal anastomosis; and, in CD, previous intestinal or perianal surgery and active perianal disease [38].

## 4. What Lessons Can Be Drawn Regarding the Use of JAKi Employed in IBD in Immune-Mediated Extraintestinal Manifestations of IBD

### 4.1. Immune-Mediated Joint Disorders

Rheumatological immune-mediated diseases, such as rheumatoid arthritis, systemic lupus erythematosus, ankylosing spondylitis and psoriatic arthritis, typically affect individuals of reproductive age; therefore, these conditions have historically had a significant impact on the health of patients and their offspring [107]. Indeed, in patients with rheumatological autoimmune diseases, complications during pregnancy are frequently observed, with an increased risk of preterm birth, preeclampsia, emergency caesarean section and low birth weight [107]. The advent of new disease-modifying antirheumatic drugs has profoundly changed the outcome and management of this delicate class of patients, on the one hand significantly improving disease control and creating favourable conditions for a regular course of pregnancy, and on the other necessitating continuous updates to ensure the safe use of these medications during pregnancy and breastfeeding, given their potential teratogenicity. These observations may be partially explained by the dysregulation of cytokine-mediated pathways, including JAK–STAT signalling, which plays a key role in immune tolerance and vascular adaptation during pregnancy (see Section 2).

JAKi, such as TOFA or UPA, represent a class of drugs that in recent years has found widespread use in the field of immune-mediated rheumatological diseases and in the treatment of IBD [108]. Given their mechanism of action and their ability to cross the placental barrier, their use during pregnancy is not recommended [107]. Therefore, the limited data available on the safety of these drugs in pregnancy derive predominantly from case reports or case series of unplanned pregnancies occurring during treatment with them.

In a study conducted by Abolhassani et al. [109], data published in VigiBase, the global pharmacovigilance database of the World Health Organisation, were analysed for reports related to the use of JAKi during pregnancy. The study identified 163 cases of women exposed to the drug during pregnancy, with a total of 213 adverse events (AE) reported. Spontaneous abortion was the most frequently reported AE, with a total of 78 cases corresponding to 47.9% of the recorded AE; among these, in 35 cases, patients were co-treated with another drug, specifically 17 were receiving concomitant methotrexate, 1 mycophenolate, while the remaining patients were not taking drugs with abortifacient potential. A total of 43 congenital anomalies were recorded across 26 isolated cases, and in no instance were organ-specific anomalies with a typical pattern observed. Complications during pregnancy were likewise highly heterogeneous, with gestational diabetes representing the most frequent cause. Furthermore, 15 preterm births were reported, corresponding to 9.2% of the adverse events described, along with 10 neonatal adverse events and 8 cases of adverse foetal outcomes, with plagiocephaly and hydrocele being the most frequent events. From a mechanistic perspective, these findings should be interpreted in light of the role of JAK–STAT signalling in decidualization and embryo implantation, suggesting a potential biological link between pathway modulation and early pregnancy loss.

The analysis [109] showed that, as also reported in previous studies, the rate of miscarriage among patients using JAKi during pregnancy was comparable to that observed in the general population; moreover, in the majority of miscarriage cases, patients were receiving concomitant treatment with known abortifacient drugs, such as methotrexate. The study sought to establish a relationship between congenital malformations and the use of JAKi; however, no specific, describable malformations were identified, and outcomes proved highly heterogeneous, underscoring the complexity of the issue and the need for further updates in this regard. No increase in the rate of term deliveries was observed in this study when compared with associations involving other drugs used during pregnancy as documented in VigiBase.

In addition, Clowse et al. [110] assessed pregnancy outcomes identified up to April 2014 from randomised controlled trials (RCTs) of tofacitinib in rheumatoid arthritis and psoriatic arthritis, as well as from post-approval non-interventional studies. The data concerned 9815 patients, of whom 1821 were women of childbearing age. Among these, 47 patients became pregnant. These patients were receiving heterogeneous treatment regimens, predominantly tofacitinib monotherapy, although some patients with rheumatoid arthritis were treated with combination therapy including methotrexate. No foetal deaths were recorded; however, certain AE were observed, including one case of congenital pulmonary valve stenosis, seven spontaneous abortions and eight treatment discontinuations. In the majority of cases, therefore, healthy neonates were born [110].

Regarding UPA, a similar study was conducted by Mahadevan and colleagues [81], evaluating cases of intrauterine exposure to upadacitinib derived from clinical trials and post-marketing case reports within the AbbVie safety database up to April 2023, and identified 128 maternal exposures. In clinical trials, among 80 reported pregnancies, 54% did not experience any AE. In the remaining cases, spontaneous abortions were recorded in 24%, elective terminations in 21%, ectopic pregnancies in 1%, and one case of atrial septal defect was observed. Conversely, in the nonclinical trial setting, out of a total of 48 pregnancies, 46% occurred without adverse events, while spontaneous abortions were reported in 38%, elective terminations in 15%, and ectopic pregnancies in 2%. Following comparison of these data with those of the general population and of patients with immune-mediated diseases, the authors concluded that no clear differences emerged, while emphasising the need for further evidence.

Some data, albeit within significant intrinsic limitations, may also be derived from isolated case reports. The first report [111] concerns a 40-year-old active smoker receiving tofacitinib at a dose of 10 mg daily for psoriatic arthritis who became pregnant. The patient had previously been treated with methotrexate, golimumab and etanercept before switching to TOFA. In agreement with the patient, it was decided to discontinue TOFA and to continue only conventional topical therapies, confirming her intention to proceed with the pregnancy. First-trimester screening indicated a low risk of Down syndrome and Edwards syndrome, and all foetal growth parameters were within the normal range: 390 g in the first week and 2500 g at the thirty-fourth week. At the end of the second month of pregnancy, a possible amniotic band was detected, which completely resolved by the fifth month; limb development was also normal. Owing to preterm rupture of membranes, the patient underwent emergency caesarean section at the thirty-sixth week, delivering a male infant weighing 2515 g and measuring 48 cm, with normal Apgar scores of 9 and 10 at 1 and 5 min, respectively. Postnatal diagnostic assessments did not reveal any congenital malformations or functional abnormalities.

A further available case [112] concerns a patient with SAPHO syndrome (i.e., synovitis, arthritis, pustulosis, hyperostosis and osteitis), who was receiving tofacitinib at a dose of 10 mg daily and discovered that she was at the fifth week of gestation after ten weeks of treatment, which was promptly discontinued upon confirmation of pregnancy. During gestation, all appropriate monitoring assessments were performed, documenting normal foetal development. At the thirty-ninth pregnancy, the patient delivered a healthy infant by caesarean section. At birth, the child weighed 2900 g, within the normal range, and measured 51 cm in length, with no congenital anomalies or functional abnormalities detected on postnatal examination.

### 4.2. Immune-Mediated Skin Disorders

Further data on the use of JAKi during pregnancy can be derived from studies conducted in the setting of immune-mediated skin diseases, which are often associated with IBD as extraintestinal manifestations.

Some data may indeed be derived from the work of Haag et al. [113] on the use of JAKi in the treatment of moderate-to-severe atopic dermatitis. Among the documented cases receiving treatment with abrocitinib, seven pregnancies were reported, resulting in three live births in good health, one ongoing uncomplicated pregnancy, two spontaneous miscarriages, and one outcome not reported. In clinical trials of baricitinib and in its post-marketing surveillance, of the 58 pregnancies identified, 12 resulted in full-term deliveries without complications and with healthy neonates, and 25 were still ongoing at the end of the study. At the same time, 10 spontaneous miscarriages were recorded, accounting for 17 per cent of the total, five elective terminations of pregnancy, two unknown outcomes, and one intrauterine death, representing 1.7% of the total, which occurred in a patient receiving concomitant methotrexate therapy [113]. These data suggest that the rates of miscarriage and intrauterine mortality are comparable to those observed in the healthy population of the United States [113]. This observation is consistent with the complex and not fully understood role of JAK–STAT signalling in early pregnancy, where it contributes to the balance between pro-inflammatory and tolerogenic immune responses. However, as in this case, the limited number of cases examined and the presence of confounding factors, such as concomitant methotrexate use and pregnancies still ongoing at the end of the study, do not allow firm conclusions to be drawn regarding the use of JAKi during pregnancy.

## 5. Available Evidence on the Use of JAKi in IBD

Despite the inherent limitation that, to date, there are no high-quality RCTs capable of fully detailing the safety profile of JAKi in the setting of pregnancy, initial evidence is nevertheless beginning to emerge.

A multicentre study has recently evaluated the transplacental transfer of UPA during pregnancy as well as its relative level of excretion in breast milk, in a small sample of treated IBD women and in forty breast milk samples analysed with mixed dosing regimens between induction and maintenance, demonstrating significant transplacental transfer and excretion into breast milk (UPA detected in all milk samples, with a peak concentration approximately 4 to 5 h after oral intake and extreme values ranging from 9 to 876 µg/L, with marked interindividual variability) [114]. Despite this, the median relative infant dose (RID) was 2.66%, below the acceptability threshold of 10% (while acknowledging that, in the absence of robust safety data in the perinatal period, this finding cannot be considered sufficient) [114]. This is particularly relevant considering that JAK–STAT signalling is actively involved in placental and foetal development, raising concerns about potential interference with these processes following in utero exposure.

Similarly, for TOFA, placental transfer has been evaluated using an ex vivo dual-side human placental perfusion model on term human placentas with isolated cotyledons, assessing drug transfer through two closed circuits, maternal and foetal, demonstrating a rapid and extensive passage of TOFA across the placental membrane with potential foetal exposure [115]. Such findings further support the biological plausibility of foetal exposure affecting pathways involved in cellular proliferation, differentiation, and immune regulation, all of which are mediated by JAK–STAT signalling (see Section 2).

This underpins the concerns driving the need for high-quality evidence on the safety of JAKi during pregnancy and breastfeeding. Moreover, as previously anticipated, given the potential adverse maternal and foetal outcomes that may result from uncontrolled disease activity during pregnancy, and considering that a proportion of patients have unfortunately already been exposed to multiple therapeutic lines, particularly those considered safer in pregnancy, with refractory disease, there emerges a need to also draw upon novel mechanisms of action [116]. Among these, JAKi are certainly extremely interesting candidates, particularly because of their rapid onset of action, which is highly desirable in an emergency setting such as a severe flare during pregnancy and is markedly superior to that of other biologics in both UC [117] and, to a lesser extent, in CD [118]. However, this potential benefit must be carefully balanced against the theoretical risk of disrupting physiological JAK–STAT-mediated processes essential for pregnancy maintenance. Moreover, this is undermined by evidence of facile transplacental transfer of JAKi (i.e., UPA and TOFA, as previously described), which necessitates high-quality safety data on foetal exposure to these drugs.

At present, large-scale studies assessing pregnancy outcomes associated with JAKi are not available, although some are currently planned, such as the DUMBO registry-derived study [119], which is expected to be completed around 2028; nevertheless, multiple case reports are available, predominantly in the UC setting and in complex, multi-failure cases.

Despite these initial data, the reported outcomes, which are mainly favourable, remain sparse and heterogeneous, precluding the formulation of specific operational recommendations and necessitating a cautious, case-by-case evaluation by the clinician in extremely complex scenarios where alternative therapeutic options are unavailable.

### 5.1. Tofacitinib

During the TOFA development programme in UC, namely the three OCTAVE RCTs, 15 pregnancies were recorded in mothers exposed to this JAKi, with spontaneous abortions reported in 13.3% of cases [80,120,121]. Although these rates appear comparable to those observed in the general population, the potential impact of JAK–STAT inhibition on early implantation processes cannot be entirely excluded.

In the context of single-case reports, some notifications have been described. For example, Mitrova et al. [122] reported the case of a pregnant woman under forty years of age with UC treated with dual therapy (i.e., vedolizumab and TOFA), which was continued with a reduction in the TOFA dosage from 20 mg per day to 10 mg per day starting from the 28th week of gestation, without particular complications during pregnancy, resulting in the delivery of a healthy newborn with normal development and growth, with follow-up of up to 15 months of life showing no signs of immune dysfunction or impaired vaccine responses.

Within the field of TOFA, data from the Spanish DUMBO registry have been reported regarding two patients who were exposed to TOFA during pregnancy [123]. The first patient, a 24-year-old woman with UC, was exposed during the first 5 weeks of pregnancy, after which TOFA was discontinued and switched to adalimumab, with no complications observed in the newborn following an uncomplicated vaginal delivery at the 40th week [123]. The second case involved a 39-year-old woman with UC who was exposed to TOFA until the 18th week of gestation and then discontinued due to worsening disease activity, with subsequent threatened miscarriage, premature rupture of membranes, and preterm delivery at 33 weeks of a male infant with polydactyly and transient apnoea and bradycardia, who was discharged without sequelae (although a causal relationship with the foetal anomalies observed was not clearly established) [123].

Rowen et al. [124], moreover, described the case of a 28-year-old woman with UC previously treated with ustekinumab optimised to 90 mg every 4 weeks, which had induced pre-conceptional endoscopic remission. However, the patient developed a disease flare at the eighth week of gestation, with the onset of steroid dependence and the initiation of treatment with TOFA 20 mg per day (after an initial failed attempt at intravenous reinduction with ustekinumab) during the second and third trimesters, achieving steroid-free clinical remission at 34 weeks, followed by caesarean section at 39 weeks and the delivery of a healthy newborn.

Finally, Arzivian et al. [125] reported a 20-year-old woman with long-standing left-sided UC who had previously been treated with azathioprine, anti-TNF agents (discontinued due to severe allergic reactions), and vedolizumab, which was stopped because of difficulties in obtaining venous access, and who developed a severe steroid-refractory flare in the pre-pregnancy phase, treated with TOFA, with initiation of combined oral contraception. Despite the latter, the patient experienced an unexpected unplanned pregnancy with exposure to TOFA until the sixth week of gestation, followed by immediate discontinuation of TOFA and switching to intravenous and subsequently subcutaneous vedolizumab, resulting in a spontaneous vaginal delivery at 37 weeks of a healthy newborn, who only developed transient neonatal jaundice, resolved with phototherapy.

### 5.2. Upadacitinib

Geeganage et al. [126], in the context of UPA-related reports, described the case of a 34-year-old woman with left-sided UC, previously failing anti-TNF and anti-IL-12/23 therapies, who, during her fourth pregnancy, developed a clinically severe flare at the 26th week with severe endoscopic activity. After hospitalisation and intravenous steroid therapy, at the 28th week, UPA was initiated as rescue therapy at a dosage of 45 mg/day, resulting in rapid clinical and biochemical remission with reassuring foetal monitoring. The pregnancy ended with a vaginal delivery at 40 weeks, after induction for oligohydramnios, with the birth of a healthy male infant (3940 g, Apgar 8/9), without skeletal or cardiac anomalies and with growth and development reported as usual during follow-up.

A further case report by Coman et al. [127] described a 34-year-old woman with long-standing severe ileocolonic CD with a stenosing and fistulising phenotype, with multiple failure to conventional immunosuppressants (thiopurines and methotrexate) as well as to six biologics including anti-TNF agents, vedolizumab and risankizumab, who had already undergone multiple intestinal surgical procedures and had residual short bowel syndrome with severe malnutrition requiring long-term home parenteral nutrition. The patient experienced an unintentional conception while on UPA treatment and developed a subocclusive episode during pregnancy at the 30th week, which was managed conservatively, with the pregnancy carried successfully to term and the delivery of a healthy newborn by operative caesarean section at 38 weeks. This favourable outcome may reflect the complexity and redundancy of cytokine signalling pathways during pregnancy, despite the theoretical involvement of JAK–STAT signalling in placental development.

As a final case report, Urquhart et al. [128] described a 30-year-old woman with UC treated pre-pregnancy with vedolizumab optimised every 6 weeks and subsequently intensified to every 4 weeks, which proved ineffective due to severe endoscopic activity, with the onset of pregnancy during active disease. The patient was treated with infliximab (5 mg/kg every 4 weeks) without response, followed by rescue therapy with UPA, achieving steroid-free clinical remission at 31 weeks, with spontaneous vaginal delivery at 35 weeks and discontinuation prior to breastfeeding, with switching back to infliximab.

## 6. Clinical Implications, Current Gaps, and Future Directions for JAKi in Pregnancy

Ultimately, as already extensively outlined in this discussion, incorporating JAKi into the therapeutic sequencing of pregnant patients raises considerable concerns given the limited data currently available and, therefore, current guidelines clearly contraindicate their use. Moreover, in addition to this, a contraindication also exists during breastfeeding because of the absence of robust data providing reassurance regarding their use [12,121,129,130]. It follows that both observational studies and RCTs are mandatory in order to enable the formulation of guideline recommendations for the management of these molecules in such settings, also taking into account the objective and ethical difficulties in conducting RCTs, both due to the existence of safer alternatives during pregnancy and the challenge of achieving large sample sizes consisting of patients who experience disease flares during pregnancy and who are already multi-failure to all safer therapeutic options.

The scope for clinical decision-making in which physicians may nevertheless find themselves includes scenarios of unexpected and unplanned pregnancy occurring during ongoing treatment with JAKi in patients with active disease, as well as scenarios of pregnancies that are both unanticipated and characterised by significant, unforeseen disease flares, in patients with a history of multi-failure to all therapeutic alternatives considered safer during pregnancy. In these scenarios, which are in some respects particularly challenging, it is likely mandatory that the patient be managed in highly specialised IBD centres and by clinicians with extensive expertise in the management of hard-to-treat IBD, in order to accurately assess risk–benefit profiles and implement appropriate monitoring strategies in conjunction with the gynaecological–obstetric team.

All of the above, particularly in cases such as acute severe ulcerative colitis arising during pregnancy, which significantly increases both maternal and foetal mortality and morbidity, and in which the only currently recommended therapeutic options, owing to their rapidity of action and efficacy profiles, are infliximab and tofacitinib [11,131,132]. It is indeed well established that prompt and effective treatment of acute severe ulcerative colitis is associated with improved maternal and foetal outcomes [133].

In these scenarios, indeed, it is extremely challenging to establish clear operational boundaries and, therefore, a case-by-case approach is essential within a multidisciplinary gastroenterological, surgical, and obstetric–gynaecological team, considering the patient’s preferences and wishes.

To compensate for the intrinsic difficulty of conducting RCTs in these scenarios, several research priorities emerge:

1. The development of large scale, multicentre, international prospective and retrospective disease specific registries, in order to acquire observational data across all intentional and unplanned JAKi exposure scenarios during both pregnancy and breastfeeding;
2. The collection of data, within the settings outlined above, particularly in the most complex disease conditions, such as acute severe ulcerative colitis, clinically significant stricturing CD, fistulising CD, and similar conditions, in order to better inform clinicians in intrinsically complex scenarios in which evidence is often lacking even outside the pregnancy setting;
3. Further generation of pharmacodynamic and pharmacokinetic data aimed at quantifying and more accurately assessing the transplacental transfer of JAKi, both in cord blood and in breast milk excretion, in order to better quantify the risks associated with such exposures;
4. The extension of long-term follow-up of offspring exposed to JAKi, in order to assess not only early neonatal outcomes, as reported in the few studies and case reports currently available, but also later outcomes, such as growth disorders, infectious risk, neurocognitive development, and others;
5. A careful weighting of risk-benefit profiles in published studies, with accurate profiling of exposed patients in order to better identify those populations in which adopting choices such as JAKi is likely to be more ethically justifiable, for example, patients with highly severe disease during pregnancy and a history of multiple therapeutic failures;
6. The further development and promotion of shared frameworks and networks among international IBD centres, possibly promoted by the major international societies and stakeholders, in order to make all of the above more feasible and more easily implementable.

**Figure 1:**
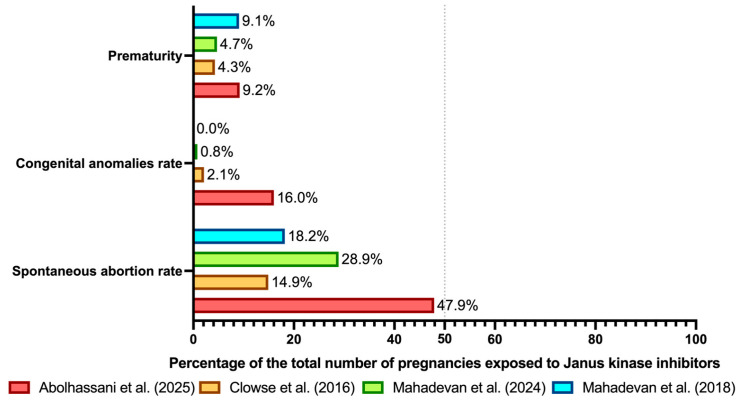
Summary figure of the rates of spontaneous abortion, congenital anomalies, and prematurity in patients with immune-mediated diseases exposed to Janus kinase inhibitors during pregnancy. These studies were not all specifically conducted in IBD, as already stated in the text of the present article. These data should be interpreted with great caution, considering this figure as a generic overall overview of the topic and bearing in mind the lack of robust studies comparing these rates with those already described in the general control population, irrespective of exposure or non-exposure to biologics and/or small molecules during pregnancy. The sample sizes for these comparisons were 163 for the study by Abolhassani et al. [109]; 47 for the study by Clowse et al. [110]; 128 for the study by Mahadevan et al. [81] (2024); and 11 for the study by Mahadevan et al. [80] (2018), respectively. Additional studies already included in this review were not incorporated into the figure because some of the reported outcomes were incomplete, inadequate, or unavailable. Case reports were not considered for obvious reasons related to the fact that rate calculations are not applicable to individual cases. The vertical dashed line indicates 50% of the sample considered.

Figure 1 summarises the available pregnancy-associated outcomes derived from the studies with the largest sample sizes.

## 7. Conclusions

The management of an IBD pregnant patient who develops a flare requiring advanced medical therapy beyond conventional treatment, or of a patient who experiences an unplanned pregnancy while receiving a drug considered unsafe in pregnancy and has active disease, represents two highly frequent and equally challenging clinical scenarios. Current evidence regarding the use of JAKi in pregnancy among patients with IBD remains, notwithstanding the above considerations, extremely limited, markedly heterogeneous, and derived predominantly from low-grade evidence such as case series or observational and pharmacovigilance registries. The establishment of dedicated prospective registries and large-scale observational studies, given the intrinsic difficulty of conducting ad hoc RCTs in scenarios such as those described, therefore becomes an essential priority in order to more precisely define both the safety of these small molecules during pregnancy and breastfeeding and their potential teratogenic risk profiles. Gastroenterologists may often encounter difficult situations in which IBD can relapse, even severely, during pregnancy in patients who have already failed mechanisms of action considered safe.

## Figures and Tables

**Table 1:** Drugs considered to be at low risk, or presumed as such, for use in patients with inflammatory bowel disease during pregnancy.

Drug	Profile Risk	References
5-ASA derivatives (i.e., mesalazine, sulfasalazine)	Low	[11,12,39,86,87,88,89,90]
Steroids	Low	[11,12,39,84,85,91,92,93,94,95]
Thiopurines	Low	[12,39,82,83,96]
Metronidazole/ciprofloxacin	Low but avoid ciprofloxacin in first trimester	[11,12,39,65,68,97]
Ciclosporin	Low (low quality evidence)	[11,12,39,98,99]
Anti-TNF	Low	[11,12,39,100,101,102]
Vedolizumab	Low (low quality evidence)	[11,12,39,100,103,104]
Ustekinumab	Low (low quality evidence)	[101,105,106]

5-ASA: 5-aminosalicylic acid.

## Data Availability

No new data were created or analyzed in this study. Data sharing is not applicable to this article.

## Appendix A

This review is based on a narrative design. The selection of studies consistent with the stated aims of this review, namely the identification of evidence regarding the use of JAKi in scenarios involving patients with IBD during pregnancy, was conducted across three main databases, namely MEDLINE, EMBASE, and, finally, Web of Science.

To perform this, the following search criteria and operators were employed:

- MEDLINE: (pregnancy OR prenatal OR maternity OR childbearing) AND (tofacitinib OR filgotinib OR upadacitinib OR JAK OR Janus kinase inhibitors);
- EMBASE: (‘pregnancy’ OR ‘prenatal’ OR ‘maternity’ OR ‘childbearing’) AND (‘tofacitinib’ OR ‘filgotinib’ OR ‘upadacitinib’ OR ‘JAK’ OR ‘Janus kinase inhibitors’);
- Web of Science: ((ALL = (pregnancy) OR ALL = (prenatal) OR ALL = (maternity) OR ALL = (childbearing)) AND (ALL = (tofacitinib) OR ALL = (filgotinib) OR ALL = (upadacitinib) OR ALL = (JAK) OR ALL = (Janus kinase inhibitors)).

No filters or inclusion and exclusion criteria were applied *a priori* to these studies, as this is not a systematic review or a meta-analysis, nor were any exclusions adopted, for example, those related to specific languages or study designs. Finally, no temporal restrictions were applied to the selection of studies. The most recent update window for this review is 30 March 2026.
